# Impact of Paternal Age on Seminal Parameters and Reproductive Outcome of Intracytoplasmatic Sperm Injection in Infertile Italian Women

**DOI:** 10.3389/fendo.2019.00035

**Published:** 2019-02-13

**Authors:** Mariagrazia Gallo, Emanuele Licata, Caterina Meneghini, Alessandro Dal Lago, Cristina Fabiani, Marcello Amodei, Domenico Antonaci, Donatella Miriello, Roberta Corno, Carmelina Liberanome, Francescantonio Bisogni, Gemma Paciotti, Carlo Meneghini, Rocco Rago

**Affiliations:** ^1^Physiopathology of Reproduction and Andrology Unit, Sandro Pertini Hospital, Rome, Italy; ^2^Science Department, Roma Tre University, Rome, Italy

**Keywords:** paternal age, ART, ICSI, infertility, sperm quality parameters

## Abstract

**Background:** We conducted a retrospective study on a cohort of couples attending the Department of Andrology and Reproductive Physiopathology at Sandro Pertini Hospital in Rome for Intracytoplasmatic Sperm Injection (ICSI)-assisted reproduction programs. Some of the couples included in the study underwent more than one ICSI cycle. Between January 2015 and April 2017.

**Objective:** To evaluate whether the advancing of the paternal age may have effect on the seminal parameters, thus negatively affecting the embryo formation, development and quality, as well as the pregnancy rate.

**Materials and Methods:** Five hundred and forty three ICSI cycles were performed on 439 couples undergoing Assisted Reproductive Technologies (ART). Patients were subdivided into three male and three female age groups having similar size:

Men: ≤38 years (M_I_), 39–43 years (M_II_), ≥44 years (M_III_).

Women: ≤35 years (F_I_), 36–40 years (F_II_),≥41 years (F_III_).

**Discussion and Conclusion:** Male age groups did not reveal any statistical significant differences in any age-related semen parameters. We also confirmed a statistical significant increase in the pregnancy rate of couples with older partner age difference and younger female. We found that the advanced male age increases the probability of obtaining one or no type A embryo (N_A_≤1), which was almost doubled in the M_III_ group in comparison with M_I_, suggesting a negative effect of male age on the efficacy of the reproductive outcome in terms of a reduced number of type A embryos. Such an effect does not seem related to semen parameters and may deserve further investigations.

## Introduction

In today's society, economic development and women's growing desire for professional fulfillment has increasingly led to the postponement of parenthood. It is well-known that both the quality and quantity of oocytes is depleted by advancing age. A number of studies have shown that the decline in oocytes is also associated with a reduction in fertility for over 35 years (1, 2). This absolute natural phenomenon accelerates between 36 and 38 years, thereby leading to a rise in the number of infertile women nearing the age of 40 who contacted the assisted reproduction centers with the belief that assisted reproduction technology (ART) is still very effective regardless of its age. While the biological clock determines the end of fertility in women, it does not seem to have a prominent role in men. Male gametogenesis goes on until late in life, according to theory, it enables men to father children even at advanced ages. However, spermatogenesis does undergo both minor and major changes over the years, as reported by the literature. During the 6th decade of life there may be important modifications in hormonal status, sperm characteristics, and histologic and cytologic testicular structure (3–6). At present, there are no legal or biological restrictions on the participation of older men in the assisted reproduction programs. Among the factors affecting the outcome of these techniques, attention is mainly given to female factors and a large body of scientific evidence confirms the importance of them on the reproductive outcome. By contrast, the fewer studies investigating the role played by male partners showed conflicting results (7–11). Given the above, we decided to conduct a retrospective study, from January 2015 to April 2017, on a cohort of couples undergoing assisted reproduction (ICSI). Our objective was to evaluate whether the advancing of the paternal age could have effect on the seminal parameters, thus affecting the embryo formation, development, quality, and the percentage of pregnancy rates.

## Materials and Methods

### Patients

The present study does not require specific approval of the ethics committee as it is a retrospective study requiring a simple “acknowledgment” (protocol 56773/2016) as per the regulation of the Lazio Ethics Committee 2. We carried out a retrospective study on a cohort of couples attending the Department of Andrology and Reproductive Physiopathology at the Sandro Pertini Hospital in Rome for the ICSI-assisted reproduction programs. Some of the couples included in the study underwent more than one ICSI cycle. All couples before, during and after the assisted fertilization path were also supported by a psychologist. From January 2015 to April 2017 we performed 1,181 ICSI cycles on 816 couples (1,026 transfers) undergoing ART. Couples who stopped treatment on their own or due to the risk of Ovarian Hyperstimulation Syndrome (OHSS) (191 cycles, 162 couples), couples whose male partners presented azoospermia or severe oligoasthenoteratozoospermia requiring Fine Needle Aspiration (FNA) (36 cycles, 33 couples) and couples whose female partners are needed, for therapeutic reasons, to cryopreserve all oocytes recovered during pick-up (215 cycles, 161 couples) or embryos achieved (69 cycles, 62 couples) and all female partners with a female disorder (such as endometriosis, reduced ovarian reserve, frequent miscarriages and endocrine ovulatory pathology (127 cycles, 108 couples, 117 transfers) were excluded from this study. The statistical analysis, therefore, includes 543 cycles (439 couples, 523 transfers). In order to assess whether and how male ages affects seminal parameters and reproductive outcome we further subdivided our cases into three male groups and three female age groups having similar size (Table 1).

**Table 1:** Male and female age groups used in the analysis.

**Male age groups**			**Female age groups**		
	**Range**	***N***		**Range**	***N***
M_I_ ≤38	25–38	174	F_I_ ≤35	24–35	141
M_II_ 39–43	39–43	186	F_II_ 36–40	36–40	210
M_III_ ≥44	44–64	183	F_III_ ≥41	41–47	192

### Examination of Seminal Fluid

All patients underwent seminal fluid examination as described by Zerbinati et al. (12) in accordance with the World Health Organization (WHO) (13) standard protocols.

### Ovarian Stimulation Protocol

All the female partners completed an ovarian folliculogenesis stimulation protocol with menopausal human gonadotropins, ultrapurified urinary Follicle-Stimulating Hormone (FSH), recombinant FSH and Corifollitropin alfa from day 2 of their menstrual cycle combined with a Gonadotropin Releasing Hormone (GnRh) antagonist from day 6. The initial dosage of gonadotropins was customized for each patient and then varied during stimulation depending on the ovarian response. When the follicular diameter reached 18–20 mm, human Chorionic Gonadotropin (hCG) 10,000 IU was administered subcutaneously. Transvaginal Oocyte Retrieval (TVOR) was performed 36 h after hCG administration. Luteal phase support was performed with progesterone by subcutaneous administration at 50 mg/day (Pleyris) or vaginal delivery at 600 mg/day (Prometrium, Progeffik), from pickup day to at least the pregnancy test, which was usually scheduled 12 days after the transfer. MII oocytes were used for ICSI. Embryo transfer was performed 3 days after oocyte retrieval. The blood sample for the pregnancy test [β-subunit of human Chorionic Gonadotropin (βhCG) assay] was scheduled 12 days after the embryo transfer. βhCG was monitored until the gestational chamber was visible on ultrasound.

### Evaluation of Fertilization, Embryo Quality, and Embryo Transfer

Fertilization was evaluated 18 h after ICSI and was considered normal when two distinct pronuclei were evident (14). Embryos were evaluated by invertoscope (Nikon Eclipse TE-2000-U) and the following parameters for the different cleavage stages were recorded: number of blastomeres, blastomere symmetry, the percentage of fragmentation, the presence of multinucleation (up to 96 h of clotting), inner cell mass, trophectoderm, and blastocele 120 h after cleavage. Embryos were embedded in a single culture medium (Sage 1-Step Medium with Human Albumin Solution, Sage, Denmark) in a trigas incubator at 37°C, 6% CO_2_ and 5% oxygen (G-185 Trigas, K-System). One to three embryos were transferred for each couple, depending on the patient's clinical history and the degree of embryo development on the second, third and/or fifth day.

Grade A 48 h embryos: 2–4 symmetric blastomeres, ≤10% fragmentation
Grade A 72 h embryos: 6–8 symmetric blastomeres, ≤10% fragmentation
Grade A 120 h embryos: expanded blastocysts (15).

All stages of gamete preparation and handling for both seminology and embryology were performed by a single biologist.

### Statistical Analysis

The statistical analysis was performed using GNU-PSPP 0.10.2 (www.gnu.org/software/pspp/). The relationship between couple of parameters in the whole cohort has been evaluated via the Pearson correlation coefficient r. ANOVA test was used to evaluate the statistical significance of differences among age groups. In the case of dichotomous variables such as β^+^ test and gestational pregnancies, logistic regression method was adopted to calculate the Odds Ratio (OR) and to evaluate their statistical significance (OR test).

## Results

The statistical analysis was conducted on a total of 543 ICSI cycles in 439 couples. Table 2 comprises of the sampled characteristics: the mean age of the female partners was 38 years (range 24–47); 50% (interquartile region Q1–Q3) were between 35 and 42 years old. The mean age of the male partners was 41 years (range 25–64); 50% (interquartile region Q1–Q3) were between 37 and 45 years old. In this cohort 67% of couples had primary infertility and 33% secondary infertility, roughly unchanged as a function of age classes. The prevalence of female, male, couple, or idiopathic diagnoses as a function of the type of infertility on the entire cohort have no statistical significant differences (Table 2). Patients were then divided into three age groups based on male or female (Table 1). Concerning the causes of infertility, for male age, we found for M_I_ and M_II_ groups about 30% due to male diagnosis, 35% to female, 15% to couples and 20% to idiopathic causes. The M_III_ group depicted roughly the same prevalence for female (35%) and idiopathic (20%) while the male diagnosis dropped below 20% and couple rise up to 28% (*p* < 0.01). Concerning the female age stratifications we found a significant (*p* < 0.01) progressive drop, while that of male diagnosis from 40% (F_I_) to 29% (F_II_) and 9% (F_III_). Couple (11%, 17%, 32%) and female (31%, 34%, 41%) diagnosis progressively grew with female age (*p* < 0.01) and the fraction of idiopathic causes remained around 17%. The age difference between men and women within this cohort study significantly increased with the rising of male age (*r* = 73%, *p* < 0.01). An inverse albeit weaker effect was observed for the age difference in relation to the female age (*r* = −29%, *p* < 0.01). In the male age group (M_I_), women were on the average ~1 year older (*p* = 0.04), while in male age groups (M_II_) and (M_III_) women were 2 years (M_II_) and 7.5 years (M_III_) younger (*p* < 0.001) than the men. When stratifying by female age, men were on average and always older, F_I_: 5 years (*p* < 0.001), F_II_: 3 years (*p* < 0.001), F_III_: 1.5 years (*p* < 0.03).

**Table 2:** Statistical description of the sample (543 ICSI cycles–439 couples).

**Female age**	**Years**	
**ICSI CYCLES: 543**		
Mean (Median)	38 (39)	
Range (Q_1_-Q_3_)	24–47 (35-42)	
σ (ΔQ)	4.2 (7)	
**Male age**		
Mean (Median)	41 (41)	
Range (Q_1_-Q_3_)	25–64 (37-45)	
σ (ΔQ)	5.9 (8)	
**Type of Infertility**	**Type I %**	**Type II %**
	67	33
**Diagnosis per infertility type**		
Female infertility	33	42
Male infertility	24	23
Couple infertility	23	18
Idiopathic	20	17

*Q1–Q3: first and third quartile; σ standard sample deviation; ΔQ interquartile distance. The diagnosis as a function of the type of infertility is reported in the bottom rows*.

The seminal parameters of all the 543 cycles are shown in (Table 3).

**Table 3:** Semen parameters for the male cohort.

	**Volume (ml)**	**Conc. (N/ml × 10^**6**^)**	**N/Ejac. (N × 10^**6**^)**	**Progressive motility (%)**	**Non-progressive motility (%)**	**Total motility (%)**	**Abnormal forms (%)**
**SEMEN PARAMETERS (543 CYCLES)**							
Mean	2.7	37	95.6	30	2.2	31.9	87
σ_m_	0.1	1.7	4.7	0.7	0.2	0.7	6
Range	0.1–9	0.1–250	0.2–607.5	0–60	0–15	0–60	70–100

*The standard uncertainty on the means is shown in parentheses, $\sigma_{m}=\frac{\sigma }{\sqrt{N}}$ (N indicates the sample number: 543)*.

**Figure 1:**
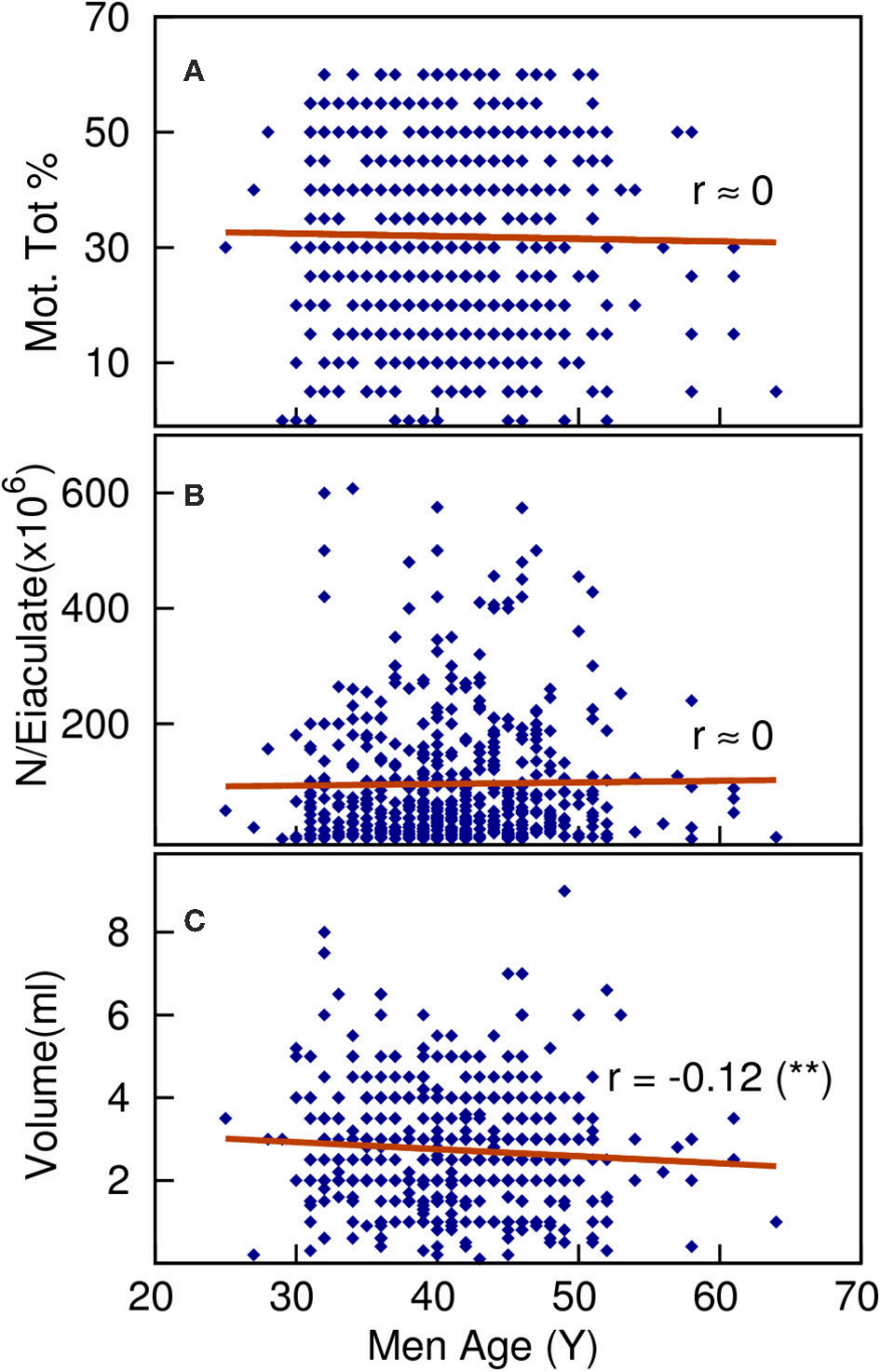
The multiple regression analysis of the whole cohort shows negligible, not statistically significant correlation between total motility **(A)** or total sperm count per ejaculated **(B)** and male age, on the contrary a weak but statistically significant negative correlation is found between ejaculated volume **(C)** and male age (*p*<0.01).

The comparison of semen parameters among the three male age groups did not reveal statistical significant difference in any of the age-related semen parameters or in the total number of cycles carried out (Table 4). The multiple regression analysis of the whole cohort shows a weak but statistically significant negative correlation (Pearson coefficient r) between ejaculation volume (V) and male age (r_V, Age_ = −0.12, *p* < 0.05) (Figure 1) or male Body Mass Index (BMI) (r_V, BMI_ = −0.15 (*p* < 0.01). Looking at the total sperm count (N) it was negatively correlated with BMI (r_V, Age_ = −0.12, *p* < 0.05) while its correlation with male age is negligibly weak and not statistically significant.

**Table 4:** Mean values, standard uncertainty of the mean values, and range for semen parameters in the 3 male age groups.

**Male Age (N.)**	**Volume (ml)**	**Concentration (N/ml × 10^**6**^)**	**N/Ejaculate (N × 10^**6**^)**	**Progressive motility (%)**	**Non- progressive motility (%)**	**Total motility (%)**	**Abnormal forms (%)**
	**Mean (σ_m_) Range**	**Mean (σ_m_) Range**	**Mean(σ_m_) Range**	**Mean(σ_m_) Range**	**Mean(σ_m_) Range**	**Mean(σ_m_) Range**	**Mean(σ_m_) Range**
**SEMEN PARAMETERS BY AGE GROUP**							
M_I_ 25–38 (174 cycles)	2.8 (0.11) 0.2–8	35.0 (3.1) 0.1–250	92.7 (8.4) 0.2–607.5	30.0 (1.3) 0–60	2.5 (0.3) 0–15	32.2 (1.3) 0–60	86.4(0.5) 65–100
M_II_ 39–43 (186 cycles)	2.7 (0.09 0.1–6	36.7 (2.7) 0.1–230	94.7 (7.6) 0.2–575	29.0(1.3) 0–60	2.1 (0.3) 0–15	31.2 (1.2) 0–60	87.4(0.4) 70–100
M_III_ 44–64 (183 cycles)	2.7 (0.11) 0.2–9	39.4 (3.2) 0.1–210	99.1 (8.7) 0.35–574	30.0 (1.3) 0–60	2.2 (0.3) 0–15	32.4 (1.3) 0–60	86.7(0.4) 70–100

The hormonal profile of male partners is shown in Table 5.

**Table 5:** Mean values of the hormonal profile.

	**FSH (UI/L)**	**Te (ng/ml)**	**LH (UI/L)**	**Inibina B (pg/ml)**
***M**_***I***_* ≤ 38 aa	4.8 ± 1.3	7.4 ± 2.3	4.2 ± 2.3	125 ± 55
***M**_***II***_* 39–43 aa	4.7 ± 2.9	6.98 ± 2.08	4.6 ± 2.7	123.5 ± 48
***M**_***III***_*≥ 43 aa	5.1 ± 1.8	7.01 ± 3.5	4.5 ± 1.09	118 ± 60

We reported data on the semen phenotype for the total male population and the population divided by age group, there was no statistical significant correlation with male age or differences between the age groups (Table 6).

**Table 6:** Semen phenotypes stratified by male age group in relation to number and percentage of ICSI cycles.

	**All**	**M_**I**_**	**M_**II**_**	**M_**III**_**
	**%**	**%**	**%**	**%**
**SEMEN PHENOTYPE**				
Normozoospermia	42	45	37	44
Oligoasthenozoospermia	31	34	29	31
Asthenozoospermia	21	14	28	18
Oligoasthenoteratozoospermia	3.5	5	2	4
Oligozoospermia	1.2	1	2	1
Asthenoteratozoospermia	0.7	1	1	1
Teratozoospermia	0.6	0	1	1

**Figure 2:**
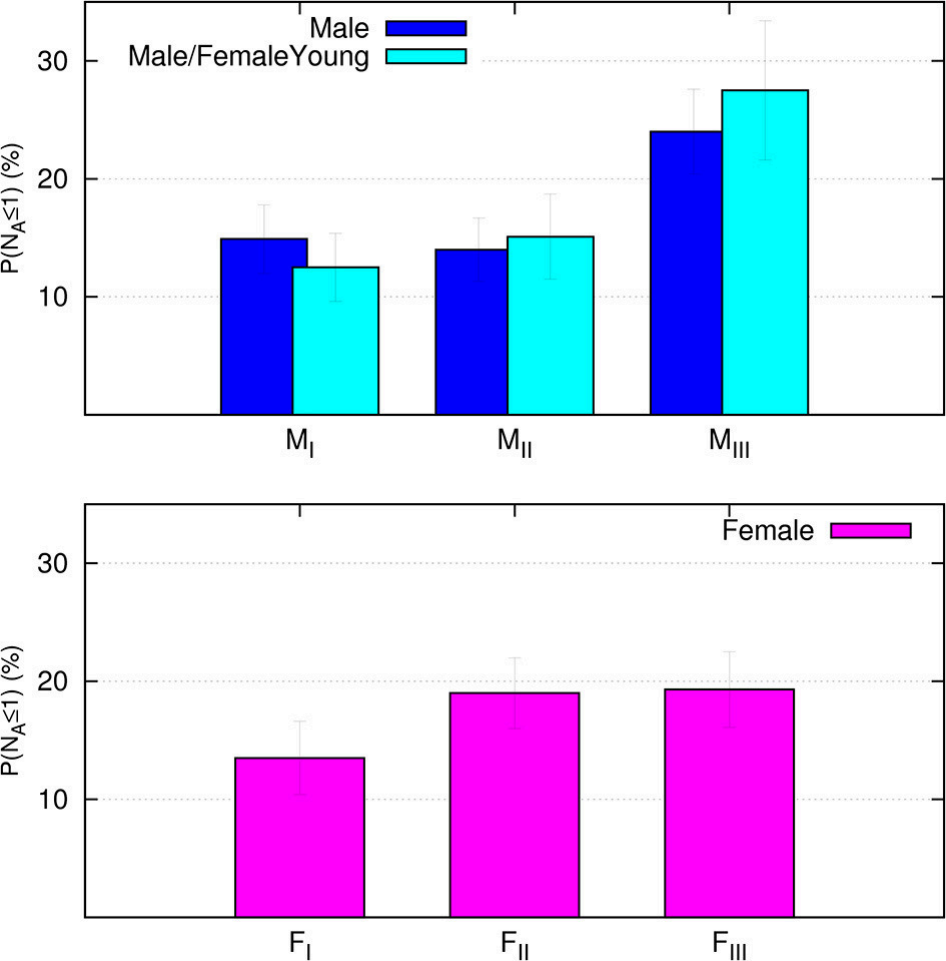
**(Lower panel)** The probability (%) of obtaining N_A_ ≤1 in the three female age bands (lower panel). The **(Upper panel)** presents the probability (%) of obtaining N_A_ ≤1 calculated in the three male age groups for the whole cohort (blue) and for a subgroup of couples including only women aged under 41 years (cyan).

The reproductive outcome of the 543 cycles are shown in (Table 7). The fertilization rate was found to be below 100% in less than the 7% of cases. No statistical significant difference between male age and reproductive outcome parameters was demonstrated. However, the chance of embryo formation was positively correlated with the percentage of progressive sperm motility (*r* = 0.1, *p* = 0.001) and negatively correlated with the percentage of non-progressive sperm motility (*r* = −0.19, *p* < 0.001). The correlation between probability of positive pregnancy test (β^+^) and clinical pregnancy (cp) have been carried out on the whole cohort as a function of women's and men's age using the logistic regression methods. We found statistical significant correlation between positive pregnancy test and woman's age, with OR_β+_ = 0.92 (the 95% confidence interval being Cl_95%_: 0.88–0.97) and OR_cp_ = 0.92 (Cl_95%_: 0.87–0.97) implying 8% year-on-year (female age) reduction of the ODDs for positive pregnancy test and clinical pregnancy. By contrast, we did not find any statistical significant correlation between male age and OR_β+_ (OR_β+_ = 0.98, Cl_95%_: 0.94–1.01) and OR_cp_ (OR_cp_ = 0.99, Cl_95%_: 0.95–1.04). The OR_β+_ has also been analyzed taking into consideration the age stratified data in female and male age groups. Taking the ODD of F_I_ and M_I_ groups as references, we found the F_II_ OR_β+_ = 0.79 (CI_95%_: 0.49–1.28) which is less than 1 but not statistically significant, and F_III_ OR_β+_ = 0.44 (CI_95%_:0.25–76) which is significantly less than 1 (*p* < 0.05). This implies that, for woman over 41-years, the ODD of a positive pregnancy test is 44% and lesser than the ODD of women younger than 36 years. Looking at the male partners the OR_β+_ are less than 1 but not statistically significant (M_II_ OR_β+_ = 0.92, CI_95%_: 0.57–1.50 and M_III_ OR_β+_ = 0.70, CI_95%_: 0.42–1.19). Therefore, the role of male age on the decreasing probability of β^+^ cannot be assessed. These findings reflect the mean couple age (Mean_age_ = 1/2 M_age_+ 1/2 F_age_): the OR_cp_ analysis as a function of the mean couple age is statistically significant, OR_tot_ = 0.96 (CI_95%_: 0.93–0.98) pointing out a 4% year-on-year reduction of the ODDs of clinical pregnancy as a function of the couple age. The effect of the age difference between the male and female partner ages (Δ = M_age_-F_age_) that gave OR_Δ_ = 1.04 (CI_95%_:1.0–1.08) pointing out the rising probability of pregnancy when the female partner is younger is noticeable. In the present study, according to Meijerink et al. (16), we used the probability of obtaining only one or no type A embryo (N_A_ ≤1) as a negative indicator to evaluate the reduced efficacy of the biological outcome. The probability of N_A_ ≤1 increases with both male and female age (Figure 2).

**Table 7:** Mean, standard deviation (σ), median, and range of the reproductive outcome of 543 ICSI cycles.

	**Mean**	**(σ)**	**Median**	**Range**
Oocytes taken	6.2	(3.5)	6.0	1–25
Oocytes inseminated	3.8	(1.7)	4.0	1–9
Oocytes fertilized	3.7	(1.7)	4.0	1–9
Fertilization rate	99%	(6%)	100%	60–100%
Total embryos obtained	3.2	(1.5)	3.0	1–9
Total embryo rate	88%	(20%)	100%	17–100%
Type A embryos	2.9	(1.5)	3.0	0–9
Type A embryo rate	90%	(23%)	100%	0–100%
Total embryos transferred	2.1	(0.8)	2.0	0–4
Type A embryos transferred	2.0	(0.8)	2.0	0–4
β^+^ test	30%			
Clinical pregnancy rate	23%			

This effect was relatively mild and not statistically significant for women but was both evident and statistically significant (*p* < 0.01) for men, ranging from around 15% or less for M_I_ and M_II_ to about 25% for M_III_. To quantify the effect of male age on the probability of obtaining N_A_ ≤1, an OR analysis was conducted. The logistic regression was carried out keeping the M_I_ ODD as a reference, and it was statistically significant OR_NA_ = 1.9 (CI_95%_:1.06–3.64) between M_III_ and M_I_ but not between M_II_ and M_I_ (M_II_ OR_NA_ = 1.06, CI_95%_:0.55–2.03). The OR_NA_ = 1.9 means that the ODD for the probability of having N_A_ ≤1 for the M_III_ males, is almost double respect to that of M_I_ males (*p* < 0.05). On the contrary for female age stratifications we found the OR_NA_ only slightly larger than 1 but non-statistically significant for F_II_ (OR_NA_ = 1.51, CI_95%_: 0.83–2.74) and F_III_ (OR _NA_ = 1.53, CI_95%_: 0.84–2.80). This finding suggests a major role of male age in increasing the probability of N_A_ ≤1. In order to further reduce the effect of correlation of male and female ages in the couple we selected a subgroup of couples including only women aged under 41 years (female age groups F_I_ and F_II_, 402 transfers) and we performed the same OR_NA_ analysis while keeping the same male age groups as before (Figure 2). We obtained M_III_ OR_NA_ = 2.66 (CI_95%_: 1.34–5.28), which is even larger than the OR_NA_ obtained considering the entire female population. This finding confirms and strengthens the negative effect of male age on the efficacy of the reproductive outcome in terms of a reduced number of A-type embryos that appears not correlated to the female age.

## Discussion

The last 40 years have witnessed a profound change in female identity, mainly due to the new role of women in the society. It is now well-known that female reproductive function after the age of 35 years undergoes a physiological aging process that far exceeds that of other organs and tissues. While men experience a gradual decline in fertility from the age of 55–65, this is not comparable with the female menopause, which marks the line between fertility and infertility and has no reproductive purpose (17). Spermatogenesis, in fact, continues until late in life and, according to theory, it enables men to father a child even at a very advanced age. However, it does undergo both minor and major changes as time passes, thereby leading to deterioration in semen parameters, hormone profile and testicular cytological structure (18). The factors affecting ART outcome is mainly related to the influence of female factors, but the few studies investigating the role of male partners offer conflicting results. Most have linked that of the male partner's to exposure to toxic substances (such as ethylene oxide, chemicals in general, solvents, and dithiocarbamates) with the risk of miscarriage (19, 20). In a study of 3,174 women de La Rochebrochard and Thonneau (7) demonstrated a clear negative effect of maternal and paternal age on the risk of miscarriage, by establishing three trends. For women aged 20–29 years, the risk of abortion is relatively low regardless of their partner's age; for women aged 30–34 years, the risk of abortion is higher if their partner is ≥40 years, and for women aged ≥35 years, the risk of abortion increases regardless of their partner's age. The authors concluded that the risk of abortion rises with the increasing age of both partners (7). Further studies have considered the effects of paternal age on the induction of premature births, although the results are inconclusive (8–11). However, the association between advanced paternal age and autosomal dominant disorders and genetic mutations has been extensively investigated (21). There is a body of scientific evidence indicating that genetic factors play an important role in reproductive timing (22). As it is well-known, the placenta is mainly of paternal origin, so if reproductive timing is guided by placental or fetal genes and if mutations in these genes occur most commonly in the gametes of older men, then advanced paternal age could play a decisive role. Zhu et al. (23) conducted a cohort study on the Danish population to investigate any association between paternal age and congenital malformations in the offspring, analyzing data from 71,937 couples between 1980 and 1996 and obtaining diagnoses of possible malformations in the firstborn of these couples from the national register. The authors concluded that men over 45 years have a 4.5-fold greater risk of having a child with trisomy 21 than men under 30 years. In the literature the association between advanced paternal age and the reproductive outcome is still under debate. As the fertilization process involves both partners, it is difficult to eliminate or control the influence of women's age on reproductive potential. To reduce the impact of female factors on reproductive potential, Frattarelli et al. (24) and Luna et al. (25) conducted studies to assess the effects of paternal age on embryonic development and reproductive outcome using donor oocytes. Both groups concluded that advanced paternal age influences the outcome of pregnancy and the percentage of blastocyst formation for men aged >50 years. Conversely, they found no statistical significant correlation between paternal age and the ability of the spermatozoa to penetrate oocyte or the formation of embryos. Luna et al. (25) also reported a statistically significant decrease in the implantation rate, but only in couples in which the male partner was more than 60 years old. Another study by Ferreira et al. (26) evaluated the effects of paternal age on reproductive outcome in 1,024 couples undergoing assisted reproduction cycles (ICSI) by investigating both normozoospermic and oligozoospermic patients. They found that paternal age negatively affects the embryo implantation and pregnancy rate in couples with a sperm concentration of <20 × 10^6^/mL. In oligozoospermic patients, the chance of achieving pregnancy dropped by 5% for each 1-year increase in age. In a review of 10 studies, Dain et al. (27) found no correlation between advanced paternal age and fertilization, implantation, pregnancy, miscarriage, and birth rates. Furthermore, no negative effect of paternal age was found on embryonic quality and stage of cleavage (days 2–3). However, there was a statistically significant decrease in the formation of blastocysts with increasing paternal age. In a review, Sharma et al. (28) found that paternal age does not significantly affect miscarriage rate or embryo quality. However, in women aged 30–34 years old, the implantation rate dropped with increasing paternal age and the pregnancy rate was significantly higher with male partners aged <30 years or 30–32 years compared to men aged 36–38 or 39–41 years. Meijerink et al. (16) conducted a retrospective study on 7,051 IVF/ICSI cycles. They did not found any statistically significant difference in pregnancy rate for men aged 35–44 years or for men ≥45 years compared to the control group of men <35 years. They also found no statistically significant effects of paternal age on embryo quality, biochemical pregnancy and spontaneous abortion, and they concluded that paternal age does not influence pregnancy rate in early IVF/ICSI cycles. In the light of literature findings, it seems evident that the influence of paternal age on the reproductive outcome is not unequivocal. The purpose of this study was therefore to evaluate whether increasing age affects sperm quality and hence the reproductive outcome. To this end, we excluded most of the possible female factors known to affect the timing and reproductive outcome (reduced ovarian reserve, endometriosis, recurrent pregnancy loss, etc.). Analysis of the couples' ages and age difference between the male and female partners revealed a positive correlation between age difference and age of the male partner, but a negative correlation between age difference and age of the female partner: the female partner was on average 1 year older than the male partner in the M_I_ age group, but younger than the male partner in the M_II_ and M_III_ classes. No statistically significant differences were found when analyzing semen parameters in the 543 cycles including after stratification by age of the male partner. From our data, we did not assess any evidence that the increasing male age may affect sperm to such an extent as to compromise semen quality, as also found by Spandorfer et al. (29). This result contrasts with some data reported in other literatures in which the authors found that semen volume, progressive sperm motility and percentage of abnormal forms were significantly lower in older men than in younger subjects (5, 30). These discrepancies demonstrate the complexity of carrying out a study that takes into consideration a significant number of subjects aged over 60 years. A further confounding factor could make the little information available on possible internal and androgenic disorders potentially affecting semen parameters in addition to physiological tissue aging. We found a statistically significant negative correlation between BMI and ejaculation volume but not on semen parameters, confirming the results of Duits et al. (31) and Shayeb et al. (32). In fact, the increase in aromatization activity caused by high concentrations of adipose tissue results in the conversion of testosterone to estrogen; as a consequence, excess leptin causes a drop in testosterone production by Leydig cells, thus altering the functionality of seminal vesicles (33). Furthermore, when stratified by male age, there was a weak but statistically significant positive correlation between the percentage of embryos formed and progressive sperm motility and a weak but statistically significant negative correlation with non-progressive sperm motility. Motility is a fundamental sperm property; its fertilizing capacity depends on chromatinic and mitochondrial integrity (34), both necessary to enable the sperm cell to swim up the female genital tract, penetrate the oocyte and form the male pronucleus. Sperm motility appears to be very important not only for natural fertility but also in assisted reproduction, especially in the most advanced technique, ICSI, which allows fertilization with very few spermatozoa. In this case it is of critical importance to have motile sperm cells, an unmistakable sign of their viability. Kasai et al. (35) demonstrated a higher fertilization and pregnancy rate in patients with higher sperm motility and mitochondrial membrane potential. It is therefore very important to understand the molecular processes underlying sperm motility, as less mobile semen samples can be treated with gene or pharmacological therapies before ART. Our results are in accordance with those of Wu et al. (36) and Begueria et al. (30) who found that paternal age did not significantly affect embryo quality, embryo cleavage stage, or miscarriage rate. However, these authors demonstrated that in women aged 30–34 years, the implantation rate dropped with advancing paternal age and the pregnancy rate was significantly higher for couples with male partners aged <30 or 30–32 years than for male partners in the 36–38 and 39–41 age groups. Concerning these parameters, we did not see a statistically significant effect of age in our data. There was a statistically significant negative correlation between positive pregnancy test and clinical pregnancy rate and age of the female partner, with an 8% year-on-year (female age) drop in the ODD ratio for the chance of getting pregnant. When stratified by female age group, our data showed that the ODD ratio for the probability of positive pregnancy test for women aged ≥41 (F_III_) is less than half of women ≤35 years (F_I_). Our results did not reveal any effect of paternal age on the probability of a positive pregnancy test and clinical pregnancy test in the total cohort sample or when stratified by male age. These results are in agreement with those found in the literature (16, 18, 24, 37, 38). When analyzing the effect on the reproductive outcome of the mean age of the couple and the age difference between the male and female partner we observed that increasing the couple age is significantly related to a reduction in the clinical pregnancy rate. We also confirmed a statistically significant increase in the pregnancy rate in couples with higher partner age difference and younger females. An important issue concerning the efficacy of the biological outcome that emerges from this study is that the probability of achieving none or only one type A embryo increases with both male and female age. This is very evident and statistically significant for the male partner indeed ODD is almost doubled in the M_III_ class in comparison with M_I_. More interestingly the negative effect of male age on raising the probability of N_A_ ≤1, is even more evident when the sample is restricted to the young women couples (female age <41 years): reducing the sample to the couples with the female partner in age groups F_I_ and F_II_, the ODD for N_A_ ≤1 probability for men in age group M_III_ is almost three time larger than M_I_. Noticeably a reduction of the quality embryo probability ODD for older men couples is also reported in Meijerink et al. (16) but without statistical significance. It is noteworthy that our finding is a relatively new result strongly supporting some negative effect of male age on the efficacy of the biological outcome, but it does not seem related to any changes in seminal parameters.

## Author Contributions

RR took part in the conception and design of the study. MG drafted the article and took part in the analysis of the data. MG and AD approved the final version of the manuscript. CarM acquired and analyzed the data. All the other authors have reviewed the manuscript critically.

### Conflict of Interest Statement

The authors declare that the research was conducted in the absence of any commercial or financial relationships that could be construed as a potential conflict of interest.
